# The role of endocrine disruptors in female infertility

**DOI:** 10.1007/s11033-023-08583-2

**Published:** 2023-07-04

**Authors:** Ana Beatriz P. Silva, Filipa Carreiró, Fernando Ramos, Ana Sanches-Silva

**Affiliations:** 1 University of Coimbra, Faculty of Pharmacy, Azinhaga de Santa Comba, 3000-548 Coimbra, Portugal; 2 REQUIMTE/LAQV, University of Coimbra, Faculty of Pharmacy, Azinhaga de Santa Comba, 3000-548 Coimbra, Portugal; 3 National Institute of Agrarian and Veterinary Research (INIAV), Rua dos Lagidos, Lugar da Madalena, Vairão, 4485-655 Vila do Conde, Portugal; 4 Center for Study in Animal Science (CECA)-ICETA, University of Porto, Praça Gomes Teixeira, 14 Apartado, 55142-401 Porto, Portugal; 5 Associate Laboratory for Animal and Veterinary Sciences (Al4AnimalS), 1300-477 Lisbon, Portugal

**Keywords:** Bisphenol A, Endocrine disruptor, Female infertility, Exposure, Phthalates, Pesticides, Dioxins

## Abstract

**Introduction:**

According to the World Health Organization, infertility is a public health problem that affects around 48 million couples and 186 million individuals worldwide. Endocrine disruptors are one of the causes that raise more concern, given that it is a problem that has evolved with the progress of society. Many chemicals are used by food industry, entering food chain, and directly affecting human health. Endocrine disruptors have the capacity of interfering with the normal hormonal action, metabolism, and biosynthesis, which can lead to a variation of the normal hormonal homeostasis. Some of these endocrine disruptors are highly associated with diseases that are positively correlated with female infertility, such as polycystic ovary syndrome, endometriosis, irregular menstrual cycle and also disturbances on processes as steroidogenesis and development of the ovarian follicles.

**Results:**

The present literature review covers various aspects of the possible relationship between endocrine disruptors and female infertility. Bisphenol A and its metabolites, phthalates, dioxins, organochlorine, and organophosphate compounds are groups of chemicals considered to have the capacity to disrupt endocrine activity and herein addressed. The results reported in in vivo studies and in clinical trials addressing endocrine disruptors and female infertility were discussed as well as their possible mechanism of action.

**Conclusions:**

Large, double-blind, placebo-controlled randomized clinical trials are needed to better understand the mechanisms of action of endocrine disruptors in female infertility, as well as the doses and frequency of exposure responsible for it.

**Supplementary information:**

The online version contains supplementary material available at 10.1007/s11033-023-08583-2.

## Introduction

Infertility is a global public health problem that affects around 8 to 12% of couples in reproductive age worldwide. According to the World Health Organization (WHO), infertility is defined as “*a disease of the male or female reproductive system defined by the failure to achieve a pregnancy after 12 months or more of regular unprotected sexual intercourse*” [1]. It has a huge impact on the population in cause since the couples can experience depression, anxiety, distress, reduced self-esteem, and a feeling of guilt and blame during the process.

There is a growing interest in the role that endocrine disruptor (EDs) can play in Public Health, namely in reproductive female health. The recent changes in the lifestyle of individuals and societies are one factor that contributes to the higher exposure to these harmful chemicals and there is a huge contribution of Endocrine-Disrupting Chemicals (EDCs) from agriculture and industrial waste and climate change. These substances are increasingly present in our environment, in our food and food packages and, in consumer products (cosmetic, daily use, etc.) and still manages to occur via inhalation of gases and air particles. Moreover, a transmission from the pregnant woman to the developing fetus or child, during gestation and lactation, through the placenta and breast milk, was also demonstrated [2].

The scientific community is quite unanimous regarding the definition of endocrine disruptors, “*an endocrine disruptor is an exogenous substance or mixture that alters function(s) of the endocrine system and consequently causes adverse health effects in an intact organism, or its progeny, or (sub)populations.*” [3]. The European Chemical Agency alongside with European Food Safety Authority (EFSA) has published in 2018 a guidance document for the identification of endocrine disruptors [4].

The present literature review covers various aspects of the possible relationship between endocrine disruptors and female infertility. The main groups of endocrine disruptors associated with female infertility are herein addressed, including bisphenol A (BPA), phthalates, dioxins and dioxin-like compounds and organochlorine and organophosphate pesticides. Finally, the results reported in in vivo studies and in clinical trials addressing endocrine disruptors and female infertility were covered as well as their possible mechanism of action.

A review very important is from *Gone et al. (2015)*. because they summarize female reproductive health, and indicate EDCs can affect the ovary, uterus, vagina, anterior pituitary, and/or steroid production, which can lead to reproductive disorders including early puberty, infertility, abnormal cyclicity, premature ovarian failure/menopause, endometriosis, fibroids, and adverse pregnancy outcomes [5].

## Endocrine disruptors

Endocrine disruptors, also called endocrine active substances, endocrine disrupting chemicals or endocrine disrupting compounds are chemicals with the ability to interfere with normal hormonal action leading to adverse health effects on an organism and/or its future generations. These abnormal activities can result in many adverse health effects since they interfere with natural hormone systems and its respective maintenance of the normal pathway of body hormones.

Endocrine disrupting chemicals, which can be found in agriculture, industry, drugs or food chain, comprise a wide variety of exogenous chemicals including synthetic compounds able to affect hormones synthesis, metabolism, and function [2]. In this work, three major groups of endocrine active substances that have a more distinguished role on female health will be explored, since they are often referred as food contaminants. Bisphenol A andphthalates, are some of the chemicals that can be found frequently in food packaging industries and can be easily identified in contamination processes. Another vast group is pesticides, more specifically organophosphate and organochloride compounds that are also very linked to adverse reproductive effects. Finally, we have dioxin and dioxin-like compounds that emerge very frequently from uncontrolled incinerations, accidental fires, and waste burning, where the principal source of human contamination is through food and through environment [6].

### Endocrine disruptors associated with female infertility

**Fig. 1:**
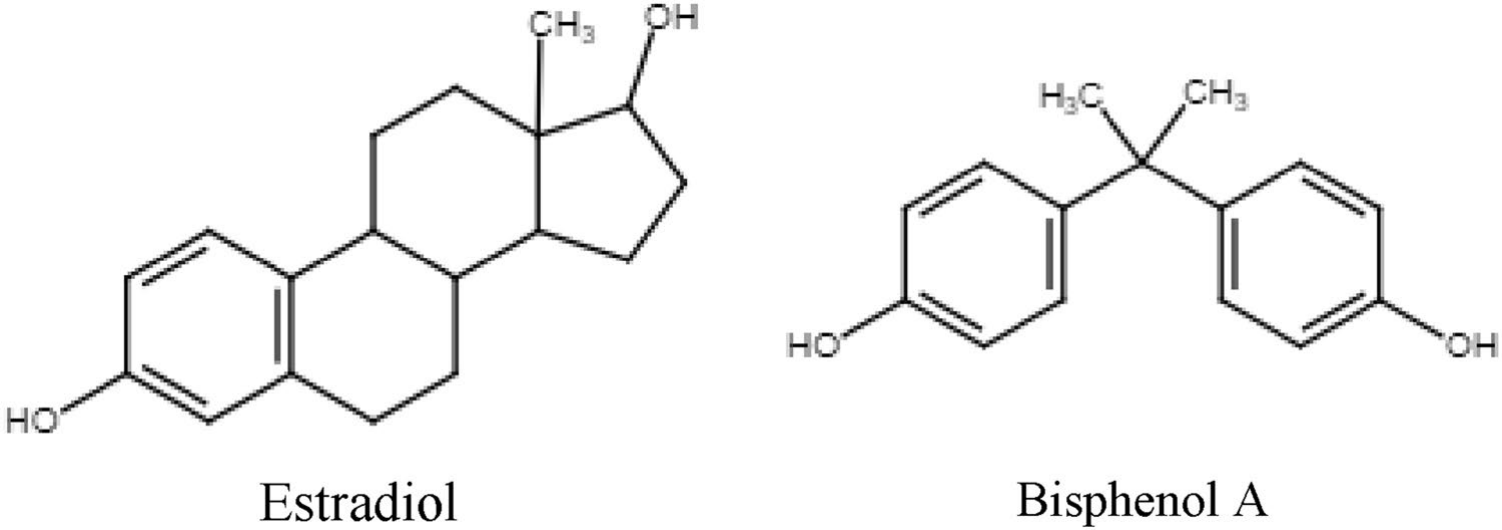
Example of an analogy of the chemical structures of the natural estrogen hormone estradiol and bisphenol A, a xenoestrogen endocrine disruptor.

EDs appear to have a role that negatively affects female infertility, examples of well-known EDs for this negative effect are present in Table 1. An increasing number of papers aim to explain and explore the aspects involving this activity and its role on reproductive health. Since the chemical structure of most EDs mimics sex gonadal hormones (e.g. Fig. 1), the reproductive system is the most vulnerable system to EDs actions. They have the ability to bind to endocrine receptors and interfere with hormonal signals, representing a threat to the normal function of the endocrine system.

**Table 1:** Classifications, chemical properties, and sources of the most common EDs

EDC	General Chemical Structure	Group	Route of exposure	Sources	Half-life
BPA		Bisphenols/plasticizers	Ingestion, inhalation, dermal absorption	Polycarbonate plastics, thermal paper, epoxy resins, plastic toys, and bottles, lining of food cans.	4–5 h
DEHP		Phthalates/ plasticizers	Ingestion, inhalation, dermal absorption	Medical devices, artciles made of PVC.	4–8 h
DDT		Organochloride/pesticides	Ingestion, inhalation, dermal absorption	Contaminated water, soil, fish.	6–10 years
TCDD		Dioxin	Ingestion, inhalation	Combustion of fossil fuels, incineration processes.	1.6–3.2 years

*BPA* Bisphenol A, *DEHP* Di(2-ethylhexyl) phthalate, *DDT* Dichlorodiphenyltrichloroethane, *TCDD* 2,3,7,8-tetrachlorodibenzo-p-dioxin, *PVC* Polyvinyl chloride

The mechanisms by which these compounds act, are still one of the major concerns to be solved. Most EDs have synergistic or antagonistic outcomes, and many can interfere with estrogen receptors (ER) or androgen receptors (AR). Aryl hydrocarbon receptor (AhR) is the most studied protein concerning its activity with EDs [7].

#### Bisphenol A

BPA is a xenoestrogen, exhibiting estrogen-mimicking, hormone-like properties and is widely present in daily life, mainly in plastic materials [8]. Among the numerous exposures possible from this compound, food exposure is the one that brings more concern, being the one that reaches the greatest number of people which can occur for a long time without being detected. It is used to produce synthetic polymers, including epoxy resins and polycarbonate, which are used in reusable bottles, kitchen utensils, varnishes and protective coatings for canned food and beverages [9]. Is one of the most investigated ECs [2].

The specific migration limit (SML) of BPA has decreased from 0.6 mg/kg food to 0.05 mg/kg food. This alteration was established on the regulation 2018/213, modifying the previous SML foreseen on the regulation (EU) N°10/2011 [10]. The main dietary source of exposure to BPA are canned food, meat products, and fish can also show high levels of BPA [9].

**Fig. 2:**
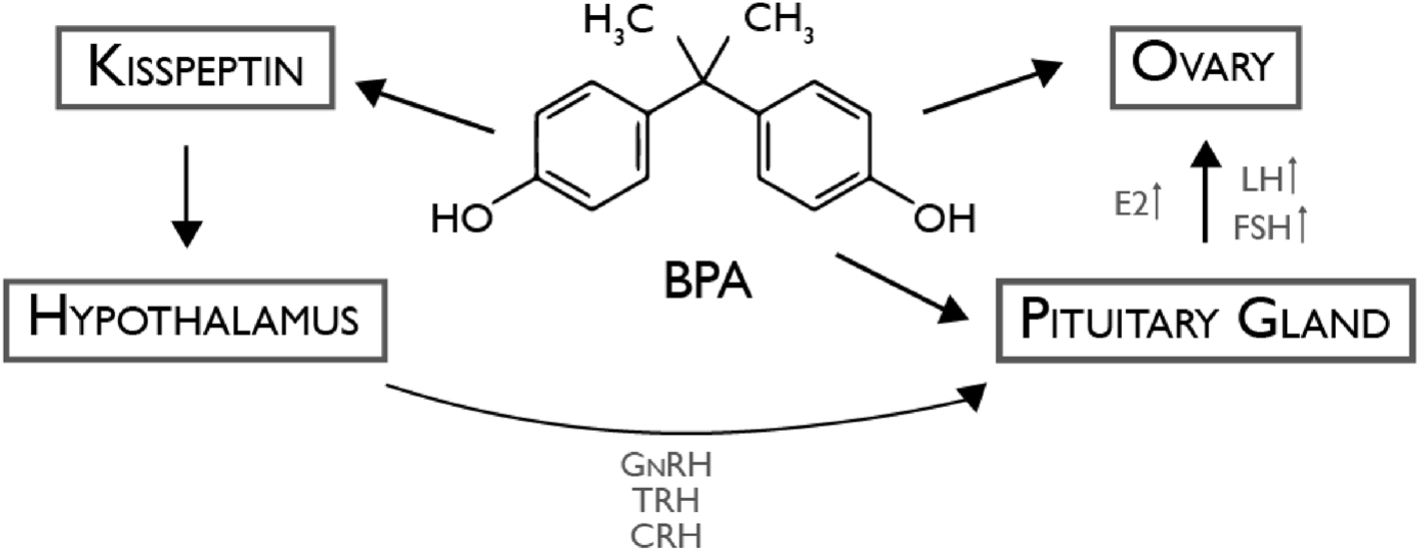
Disruptive mechanism induced by BPA on HPG axis. BPA regulating kisspeptin expression leads to alterations in GnRH levels, affecting the release of FSH, LH, and sex hormone, resulting in adverse effects on the reproductive system; *BPA* Bisphenol A, *GnRH* Gonadotropin-releasing hormone, *TRH* Thyrotropin-releasing hormone, *CRH* Corticotropin-releasing hormone, *E2* Estradiol, *LH* Luteinizing hormone, *FSH* Follicle-stimulating hormone.  Adapted from [12]

BPA can disrupt at many levels, the feedback control system including hypothalamic–pituitary–gonadal (HPG) axis is one of them (Fig. 2), being the reason why this ED is closely connected to reproductive health. On the HPG axis there is evidence that this chemical can alter the levels of Gonadotropin-releasing hormone (GnRH), regulating kisspeptin expression. This alteration traduces in the release of Follicle-stimulating hormone (FSH), Luteinizing hormone (LH), and sex hormone, which results in adverse effects on the reproduction system via irreversible impairment of the HPG axis [11].

The International Agency for Research on Cancer (IARC) has designed a system to categorize different agents and their carcinogenicity to humans. Based on this classification BPA is inserted in group 2 A- “Probably carcinogenic to humans” [13]. This designation is attributed when component lacks evidence of carcinogenicity in humans but, in animals experiments, there is strong and sufficient evidence of the carcinogenicity, and that the mechanistic in cause also runs in human organism [14].

#### Phthalates

Another group of substances used in the plastic industry is phthalates. It is estimated that 4,9 billion kilograms are the global production of this plasticizer per year [15].

Phthalates are used to make the plastic more malleable, more transparent, and with larger durability. They are considered endocrine disruptors since they have the capacity to interfere with biosynthesis, metabolism, and hormonal activity, changing the natural way of our hormonal system [15].

There is a growing concern about the role that phthalates play in fertility. Some of them had already been characterized as toxic substances for reproduction and because of that, some restrictions have been made on the industry [16]. And can disrupt follicle growth pattern, increase oxidative stress and cause follicle death [17].

It is unquestionable that the production of polyvinyl chloride (PVC) is the most common use of phthalates. PVC is an extensively used plastic around the world, and it is present in numerous products present in our daily life [14]. In plastics, phthalates are not covalently bound to the structure, which means that they leach from the products and end up contaminating food, water, soil, air, and dust [17]. Examples of that are food packaging and packaging in general, manufacture of toys, certain pharmaceutical formulations, and thousands of consumer goods [16].

Phthalates have been found in liver, lungs, urine, amniotic fluid, ovarian follicular fluid as well in adipose tissue. The presence of phthalates in ovaries and amniotic fluid means that exposure in life-long starting from germ cell development and fetal life. Occupation can be a factor leading to higher levels of exposure, for example in hairdressers’ urine phthalate levels are elevated compared to the general population [17]. Urinary phthalate levels in women have been associated to decrease rates of pregnancy, increased rates of miscarriages, pregnancy complications as well a diminished ovarian reserve [17].

A very extensive and robust study was carried out in order to analyze the presence of phthalates in food and beverages, such as those included in Table 2, and also highlights the many different toxic effects that these chemicals may perform on human health, namely the adverse effects on reproductive health. What arises from all the different analyses on the different groups of food and beverages, was that there are a lot of factors that contribute to the migration of these chemicals from packaging into food. Another conclusion was that alongside the evolution of the food chain is the increase of the phthalate’s concentration [16].

**Table 2:** Principal phthalates that had been demonstrated to interact with estrogen receptor and progesterone receptor (PR) in humans. Data extracted from [18]

Parent phthalates (Phthalate-diester)	Primary metabolites (Phthalate-monoester)
DEHP	MEHP
DiBP	MiBP

*DEHP* Di(2-ethylhexyl) phthalate, *MEHP* Mono-2-ethylhexyl phthalate, *DiBP* Di-iso-butyl phthalate, *MiBP*  Mono-iso-butyl phthalate

Di(2-ethylhexyl) phthalate (DEHP) is one example of a high-molecular-weight phthalate and one of the most studied phthalates. It is mostly used as a plasticizer in the production of PVC. This phthalate formerly had been classified as possibly carcinogenic, however, according to the most recent IARC evaluation DEHP “is not classifiable as to its carcinogenicity to humans (Group 3) because peroxisome proliferation has not been documented in human hepatocyte cultures exposed to DEHP nor in the liver of exposed non-human primates. Therefore, the mechanism by which DEHP increases the incidence of hepatocellular tumors in rats and mice is not relevant to humans” [19].

For the past years, the effects of phthalates in the male reproductive system were much more documented and explained than the effects on the female reproductive system. Nowadays is clear that phthalates play a severe role in both genders [20]. The disruptive mechanism behind this class of EDs is not fully understood, nevertheless, disorders on the HPG axis have been associated with some phthalates, which is crucial to develop a correct reproductive development [20]. The mechanism involved is still uncertain, nevertheless, studies have revealed the structure dictates the response that will be triggered, that is, it can have an inhibitory or stimulating response on the AR and ER activity. For example, it was shown that DEHP, and its respective metabolites, do not bind to AR [20].

#### Dioxins and dioxin-like compounds

The term dioxin is applied for a group of 210 chlorinated compounds which divides into two subgroups, the polychlorinated dibenzofurans (PCDFs) and the polychlorinated dibenzo-p-dioxins (PCDDs). Within these 210 compounds, the 17 congeners with chlorine atoms at the 2,3,7 and 8 positions are found to be of major importance once this characteristic gives them a more resistant metabolic degradation, which results in body accumulation [21].

Dioxins are dangerous environmental pollutants that belong to a group called persistent organic pollutants (POPs). The interest in this group, to the scientific community, has been growing due to their toxic effects on animals, the environment, and human health [22].

The main source of dioxins is anthropogenic especially due to combustion processes but can also emerge due to natural processes. The formation occurs at high temperatures between organic compounds and chlorine. Another huge source of dioxins is uncontrolled waste incinerators [23]. Although many of these releasing events of dioxins to nature are local, the spreading is global. According to the WHO “the highest levels of these compounds are found in some soils, sediments and food, especially dairy products, meat, fish, and shellfish. Very low levels are found in plants, water, and air” [22]. As mentioned, fatty food is an important source in terms of human exposure. A study conducted in Norway was performed in order to evaluate the risk-benefit of consumption of seafood, more specifically fish fillet, farmed salmon, wild mackerel, herring and spring spawning. Despite the important role as a huge supplier of docosahexaenoic acid (DHA), eicosapentaenoic acid (EPA), and vitamin D, it is also pointed out as a source of dioxins. It has been concluded that even with the high levels of EPA, DHA, and vitamin D, if the species were consumed according to the recommendations, the levels of dioxins consumed were exceeded [24].

In the field of dioxin toxicology, even though the mechanism is not fully comprehended, is clear that these compounds induce various toxicities through their tight relation with the AhR. AhR is present in ovaries tissues and performs a starring role in the regulation of ovarian follicular growth and steroidogenesis [25]. This connection between AhR and dioxins triggers all the endocrine signaling routes that are mediated by the steroid hormones [26].

2,3,7,8-tetrachlorodibenzo-p-dioxin (TCDD) was classified in 1997 by the IARC as a group 1 carcinogen. This category “is used when there is sufficient evidence of carcinogenicity in humans” [27]. A very high number of studies have proven that TCDD is a potent carcinogenic with vast mechanistic information indicating that this substance could disrupt multiple endocrine pathways through a mechanism involving AhR [28].

#### Organochlorine and organophosphate pesticides

Pesticides are synthetic molecules that emerged with the aim of killing or repelling pests, fungi, insects, diseases from plants during their growth. Their use is growing stronger since the global market for these pesticides is steadily expanding and are widely used in agriculture, consumer products, and industrial products. Pesticides have attracted international attention and have recently come to be regarded as possible contributors to reduced human fertility [29, 30].Human exposure to organophosphate pesticides is now widespread in many countries and has become a global health issue [29]. The Table 3 summarizes the most common organochlorine and organophosphate compounds, their chemical structure, use, and persistence [31].

**Table 3:** The most common organochlorine and organophosphate compounds, their chemical structure, use, and persistence. Adapted from [21]

Chemical name	IARC group	Use	Persistence in environment	WHO classification based on rat oral LD50
DDT	Group 2A	Acaricide insecticide	High persistence Half-life: 2–15 years	Moderately hazardous
DDD	–	Insecticide	High persistence Half-life: 5–10 years	Acute hazard in unlikely
DDE	–	Insecticide	High persistence Half-life: 10 years	Slightly hazardous
Diazinon	Group 2A	Insecticide	Moderately persistence Half-life: 37–38 days	Moderately hazardous

*DDT* Dichlorodiphenyltrichloroethane, *DDD* Dichlorodiphenyldichloroethane, *DDE* Dichlorodiphenyldichloroethylene

It has been studied that the long exposure to these compounds its dangerous even in very low doses, they have the power to affect the organism since that many of them play a role as endocrine disruptors [32, 33].

One specific group of pesticides, which is widely used, is organochlorine compounds (OCPs) wich can be considered as persistent organic pollutants (POPs) that are environmental pollutants and known as carcinogenic chemicals. The organophosphate insecticides were created to replace the so relentless OCPs, since they were proven to have low persistence in the environment and a rapid breakdown [34].

Dichlorodiphenyltrichloroethane (DDT) is one of the most studied substances since a huge number of hormone-related effects on wildlife have been attributed to it. It was created around 1940, initially used to combat insect-borne human diseases such as typhus and malaria. As one of the first synthetic insecticides was also effective for insect control and was extensively used during many years after that. Despite its abolishment around 1970, DDT remains relevant to living populations for many reasons, it is a persistent pollutant in the environment, so people worldwide continue to be exposed [16].

Endocrine disruptors can alter the pathway of hormones, acting as agonist or antagonist. DDT (isomers and metabolites), as an organochlorine pesticide that has reported endocrine disruptive activity, has demonstrated its dangerous activity when binding with hormone receptors, such as AR, where displays an antagonistic role, and its capacity to increase the progesterone receptor in the ovary and uterus, also raises concerns [35, 36].

This insecticide was classified, by IARC evaluation, as a group 2 A substance that is probably carcinogenic to humans. The mechanistic data provide strong support for the carcinogenicity findings of DDT [37].

#### Parabens

Parabens are widely used, as efficient preservatives in the cosmetic industry, food, pharmaceutical products, and other products that care for their use as antimicrobial agents. They are structurally similar to bisphenols, comprising many sub-classes including methyl, propyl and butyl parabens [38].

On a first approach, parabens were considered in this paper as a relevant endocrine disruptor to explore and study their possible role in female infertility. However, after a long search and study of diverse publications and studies, they were left out, because the most recent studies report that parabens are still considered safe to use since there is a lack of scientific evidence about their toxicity and endocrine disruption activity. Nevertheless, studying this class of substances is still necessary to better address the scientific gaps and disagreements existing and to provide better and more exact answers [1, 39].

## Infertility

Infertility is a worldwide problem that has a huge impact on families and communities. It is estimated that 48 million couples and 186 million individuals are affected by infertility globally. It is a disease that can affect both genders. Female infertility is a condition that happens due to disorders in the female reproductive system, such disorders can present as uterine, tubal, and ovaries disorders, and also can come from endocrine disorders that led to abnormalities of reproductive hormones [36].

“The most widely used indicator of fertility is the total fertility rate: this is the mean number of children that would be born alive to a woman during her lifetime if she were to pass through her childbearing years conforming to the age-specific fertility rates of a given year”. In 2018 the fertility rate in the Europe was calculated, 1.55 live births per woman were the result. The lowest fertility rate was 1.43 and it was registered in 2001 and while the highest rate was 1.57 in 2010 [40].

### Main causes

There are many causes pointed as responsible for female infertility. For a normal function of the reproductive system, women need to have functioning ovaries, uterus, fallopian tubes, and a normal endocrine system. When something compromises one of these systems, fertility may be jeopardized [41].

Hereupon infertility may be caused by tubal disorders, uterine disorders, among which endometrioses stand out, ovaries disorders, such as polycystic ovary syndrome (PCOS), and disorders that endanger the endocrine system and its normal function [27, 42].

Growing evidence proposes a possible connection between endometriosis and environmental pollutants, more specifically the ones with disruptive activity. Environmental pollutants, such as dioxins show strong evidence that this exposure can lead to diseases as endometriosis [43].

Besides the conditions referred above, there are many risk factors that contribute in a very sharp way, such as smoking [44], excessive alcohol drinking [45], obesity [46], and age [47]. Other curious studies reveal many other possible risks that can contribute to infertility itself, there are numerous publications about the role of vitamin D in female infertility, revealing that this vitamin is essential to the reproductive system, and without it, the fertility can be compromised [56, 57, 58]. Another interesting finding during this research was that contrary to what most women think [48], there is no evidence that the use of an intrauterine device can play a negative role in female fertility [49, 50].

## Effects of endocrine disruptors on female fertility

Endocrine disruptors have gained a lot of attention from the scientific community in the past years. A lot of laboratory and human studies have been published regarding the numerous chemicals causing this dangerous endocrine activity. The health effects that these compounds cause, have been increasing alongside the publications, proving that can be responsible for multiple diseases.

The endocrine system has an essential role in many physiological systems since it is responsible for hormonal communication, which is based on the production and release of hormones from different glands in the bloodstream, which coordinates various functions in our body by carrying messages. Hormones are essential for many processes such as metabolism, development and growth, and reproductive functions. When the hormonal imbalance is not accurate, health problems can arise due to these alterations in the number of hormones produced by the glands. Many things can affect this balance, and endocrine disruptors, as the name indicates, are one of the major responsible [51].

### Bisphenol A and phthalates

Studies performed in vivo, and some clinical trials in different populations, have been crucial to the understanding of the different ways BPA and phthalates can affect reproductive health.

The most common cause of infertility, as a result of anovulation, is PCOS and it is estimated to affect 6–9% depending on the used criteria of the National Institutes of Health (NIH) [52]. Women that suffer from this condition, present a hormonal imbalance that interferes with reproductive processes.

Some human studies (Table 4) demonstrate that elevated BPA concentrations are detected in adolescent girls, and women with PCOS, when compared to the control group, suggesting the potential role of this substance in this pathology [53]. Serum BPA level in market seller women with PCOS was evaluated, as well as metabolic and hormonal effects of this exposure, comparing to a control group, and a positive correlation has been demonstrated between the exposure and the PCOS physiopathology [54]. Another study was conducted on adolescents’ girls with PCOS, to observe the serum levels of BPA and its possible relationship with obesity. It was concluded that adolescents with PCOS had higher levels of BPA than the group control, independently of obesity, carrying again to the perspective that BPA can be part of the cause of PCOS [55]. Table 4 shows the in vivo studies with different chemicals found in food [12, 56].

**Table 4:** *In vivo* studies with plasticizers found in food

Research design	Dosage regimen	Parameters monitored	Outcomes/main conclusion of the study	References
Investigate ovarian folliculogenesis and steroidogenesis in adult female rat offspring born to mothers exposed to low doses of BPA	BPA50: 50 mg/kg day; BPA0.5: 0.5 mg/kg day	Estrous cycle; Average size of preantral and antral follicles	Folliculogenesis and steroidogenesis are targets of BPA within the ovary	[46]
Effect of a low dose of BPA on the reproductive axis of prepubertal female rats	0.1% ethanol or BPA in their drinking water	Hormone levels	LH and estradiol levels increased significantly meanwhile, FSH ones showed no significant changes. The number of primary, secondary, and atretic follicles increased and antral ones were decreased. Early exposure to a low dose of BPA disrupts the normal function of the reproductive axis in prepubertal female rats	[47]
This study had the goal to study the BPA effects on the ovaries function and structure and also to access to the levels of expression of the genes related to follicle development	10, 40, and 160 mg/kg of BPA	Serum estradiol (E2) and progesterone (P4); Rat body weights and ovary coefficients; The number of follicles at different stages; Changes in the mRNA expression of FIGLA, H1FOO, and AMH genes; Changes in protein expression of FIGLA, H1FOO, and AMH genes	This study demonstrates the probable negative role that BPA plays in ovarian development, and how the genes related to follicle development can be part of these outcomes	[57]
This study was performed to examine whether prenatal exposure to BPA analogs, BPE and BPS, negatively impacts female reproductive functions and follicular development using mice as a model	BPA, BPE or BPS (0.5, 20 or 50 µg/kg/day)	Serum levels of E2 (sensitivity 3 pg/ml) or testosterone (sensitivity 90 pg/ml)	Prenatal exposure to BPA analogs, BPE and BPS, have effects on fertility in later reproductive life probably due to the disruption of early folliculogenesis	[58]
Effects and potential mechanism of BPA on mouse ovarian follicular development and FGSCs	BPA (12.5, 25, and 50 mg/kg/day)	–	The effect of BPA on ovarian follicular development and FGSCs, especially the effect on FGSCs, suggests a novel mechanism of how BPA causes female infertility	[59]
The aim of this work was to test the effect of chronically exposed female mice to a mixture of three phthalates and two alkylphenols from conception to adulthood at environmentally relevant doses	Two doses: 1 and 10 mg/kg body weight/d of the total mixture	Plasma hormonal levels; Reproductive endpoints; Histological evaluation of the number of preantraland antral follicles; RNA extraction and real-time polymerasechain reaction; Protein extraction and Western blotting	These results indicate that not only exposure but also its level is relevant to assess the effective contribution of EDs in the development of diseases	[48]
To test whether DBP causes ovarian toxicity	DBP at 0.01, 0.1, and 1000 mg/kg/day	Estrous cyclicity; steroidogenesis; ovarian morphology; Apoptosis and steroidogenesis gene expression	A 10-day exposure to DBP disrupted reproductive processes in CD-1 mice; DBP exposure resulted in decreased circulating E2; Antral follicle numbers and apoptosis gene expression were altered at low doses; Estrous cyclicity and corpora lutea counts were altered at a high dose; DBP exposure resulted in altered steroidogenesis gene expression	[49]
Investigate the negative effects of DEHP exposure on oocyte development	DEHP (40 μg/kg body weight)	Cytoskeleton; apoptosis; ROS levels; epigenetic modifications; Protein Juno receptor	DEHP exposure reduced the maturation and fertilization capabilities of mouse oocytes by affecting cytoskeletal dynamics, oxidative stress, early apoptosis, meiotic spindle morphology, mitochondria, ATP content, Juno expression, DNA damage, and epigenetic modifications in mouse oocytes	[50]

*BPA* Bisphenol A, *LH* Luteinizing hormone,* FSH* Follicle-stimulating hormone, *E2* 17β-Estradiol, *P4* Progesterone, *BPE* Bisphenol E, *BPS* Bisphenol S, *FGSCs* female germline stem cells, *EDs* Endocrine disruptors, *DBP* Di-n-butyl phthalate, *DEHP* Di(2-ethylhexyl) phthalate

As can be seen in Table 5 [54, 55, 60, 61], a study in China found strong evidence that BPA is also disruptive to women’s hormone homeostasis, compromising reproductive health. This cross-sectional study was based on the assumption that women working in a factory of epoxy resins, which is a major source of this dangerous chemical, would be much more exposed. As expected, the group of women working in the epoxy resin factory, had significantly higher serum levels of bisphenol A than the group of workers in other factories. This study underlines the importance of assessing BPA contamination and all its concerns related to Human Health [60].

**Table 5:** Observational studies of plasticizers and fertility-related problems

Participants/subjects and research design	Patients	Patient parameters	Outcomes/main conclusion of the study	References
Investigate the role of BPA in the pathogenesis of PCOS and other metabolic parameters	112 girls with PCOS and 61 controls	Serum BPA and oral glucose tolerance test	Adolescents with PCOS presented higher BPA concentrations than controls and there was a significant relation with androgen levels	[45]
To investigate possible associations between reproductive hormone levels among woman exposed to BPA	106 women exposed and 250 unexposed	Blood samples to analyze: FSH, LH, E2, PRL and PROG; Urine samples for BPA measurement	Evidence of disruptive activity of BPA on women's hormone homeostasis were found	[51]
Evaluate serum levels of BPA in exposed women with PCOS and hormonal and metabolic effects	62 women with PCOS and 62 healthy women	Serum samples to analyze BPA; Fasting blood; Triglyceride; Cholesterol HDL and LDL; TSH concentration and LH:FSH ratio	BPA levels were higher in BPA exposed PCOS women than the group of healthy women. Major differences in the other metabolic parameters	[44]
Search the presence of eight phthalate metabolites on women attending an infertility clinic and its possible correlations	112 women	Urine samples per cycle to measure 11 urinary phthalate metabolites	DEHP and DiDP concentrations were inversely associated with oocyte yield and number of matured oocytes at retrieval; DiNP and DiDP were associated with reduced fertilization; DEHP metabolites were negatively associated with probable clinical pregnancy and live birth following IVF	[52]
Study the concentrations of 8 phthalate metabolites	112 women attending an infertility clinic	Follicular fluid and urine samples	Most of the studied phthalates were highly detected in the ovarian follicular fluid of women undergoing IVF despite in lower doses than those shown to induce ovarian toxicity in animal studies	[53]

BPA Bisfenol A, PCOS Polycystic ovary syndrome,* FSH* Follicle-stimulating hormone, *LH* Luteinizing hormone, *E2* 17β-Estradiol, *PRL* Prolactin, *PROG* Progesterone, *TSH* Thyroid stimulating hormone, *DEHP* Di(2-ethylhexyl) phthalate *DiDP* Di-isodecyl phthalate, *DiNP* Di-isononyl phthalate, *IVF* in vitro fertilization

The hypothalamic-pituitary-ovarian (HPO) axis is a complex system responsible for many processes, that must be functioning correctly in order to accomplish reproduction. If these complex interactions between the brain and the reproductive tract are disrupted, this system may alter its normal function, and lead to reproductive health problems, such as reduced fertility or even infertility itself [62].

A study, performed on female rats, showed that early exposure to small doses of BPA has a huge impact on the normal function of the reproductive axis, where there are significant changes in the hormones [63].

To investigate the effects of oral exposure to BPA on ovarian folliculogenesis and steroidogenesis, a study was conducted on adult female rats, which were exposed to different doses of environmental estrogen (BPA50 and BPA0.5). Ovaries from both BPA-treated groups showed some changes, corpora lutea numbers were increased with incomplete folliculogenesis. BPA50 group showed a reduced expression of androgen receptors at distinct stages. BPA 0.5 showed a changed expression of AR where the expression of the mRNA-follicle-stimulating hormone receptor was higher. These results came to support the idea that folliculogenesis and steroidogenesis are targets of BPA [12].

An additional study investigated the outcomes of BPA on female germline stem cells (FGSCs) and the development of mice ovaries. Female mice were administered solutions of BPA (12.5, 25 and 50 mg/kg/day). There was a slight increase in the number of follicles that had degenerated before coming to maturity, the atretic follicles. Furthermore, a reduction in the counting of primordial and primary follicles and corpus luteum was registered on higher concentrations of BPA. Another conclusion that came with this study was the suggestion that this chemical can accelerate ovaries apoptosis and promote the inhibition of its follicles [18].

Speculations on the influence that the exposure of high concentrations of BPA has on the normal development of the ovarian follicles were the starting point for this study. Pre-puberty female rats were exposed to BPA (0, 10, 40, 160 mg/kg) to investigate its effects on ovarian development, and also the levels of expression on the development-related genes. In summary, BPA exposure during this pre-puberty period may interfere with the development and function of ovaries, and gene-related processes can be behind this mechanism of BPA toxicity during the development of the ovaries [64].

The industry, when confronted with all the objections created against the use of BPA, started to use other compounds to replace it. Bisphenol S (BPS), bisphenol F (BPF), and bisphenol E (BPE) are three examples of BPA analogs, created to be inserted in the production of polycarbonates and epoxy resins. There are few studies about their endocrine activity and adverse health effects, but they raise similar concerns to BPA [64]. An example is this study performed on CD-1 mice orally exposed to different concentrations of the analogs: BPA, BPE, or BPS (0.5, 20, and 50 µg/kg/day). The beginning of puberty and irregular estrous cyclicity, particularly with lower doses, was correlated to this exposure, as well as matting problems beginning at 6 months of age. By the time of 9 months, the pregnancy rate was diminished, and some deaths at birth were reported. All of this data conducts us to the dangerousness of the analogs and BPA itself, on fertility problems that can be observed later in life [65].

Even more about an observational prospective cross-sectional study carried out in Italy, was performed in a women aged 18–40 years, 43 fertile and 110 infertile women (153 women) enrolled in 3 different areas in Italia representing different living environment scenarios, such as Roma (Lazio, Central Italy), a metropolitan city, with specific metropolitan environment and lifestyle; Ferrara (Emilia-Romagna, Northern Italy), a medium-sized town amid a prosperous area with many farms and small-sized or medium-sized industries; Sora (Lazio, Central Italy), a rural municipality characterized by intensive agricultural activities. BPA levels were detected in infertile women, 41.8% and in fertile women with 23.3%, a slightly lower value. BPA levels, in a metropolitan area, are 71.4% in infertile women and 23.1% in fertile women, in urban area are 26.3% in infertile women and 27.3% in fertile women, lastly in rural area are 4.4% infertile women and 12.5% fertile women. These values demonstrating that infertile women living of metropolitan area had a trend toward higher percentage of BPA detection compared to infertile women living urban and rural areas and that infertile women had a trend toward higher percentage of BPA detection than fertile women in metropolitan area. The mean of serum BPA levels was 10.6 ng/ml in infertile women and 4.8 ng/ml in fertile women [2]Another class of chemicals, which has been raising some concern related to the impact on female reproductive health, are phthalates. However, when compared to BPA is an impact much less studied [66]. The effects of phthalates on female reproductive health have been studied and highlighted by various in *vivo/in vitro* studies and some clinical trials too.

In this study, different doses of a mixture of three phthalates, DEHP, di-n-butyl phthalate (DBP), and benzyl butyl phthalate and two alkylphenols (1 and 10 mg/kg body weight) were administrated on female mice. This complex EDs mix was proven to modify reproductive parameters, such as the weight of the uterus and ovaries, the estrous cyclicity, levels on the reproductive hormones, and altered some features in the steroidogenesis too [67].

Another research focused on the disruptive activity of another phthalate, DBP, analyzed the effects of a short exposure (10 days) in CD-1 mice. DBP showed evidence of ovarian toxicity, where alterations on different levels of hormones, such as luteinizing hormone (LH) follicle-stimulating hormone (FSH) and estradiol (E2) were verified. Complex processes as steroidogenesis were altered by the disruption of genes involved. Future work on clarification of how this endocrine disruptor may act on the changes at the gene expression is needed [68].

**Fig. 3:**
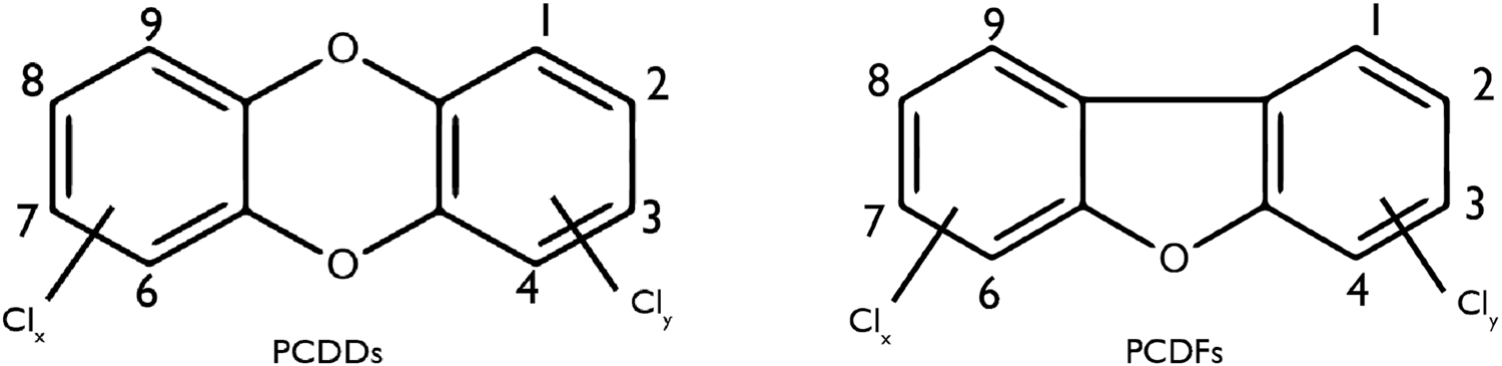
Structure of dioxins (PCDDs and PCDFs). Relevant PCDD/Fs are substituted with additional chlorines at positions 2,3,7 and 8; *PCDDs* Polychlorinated dibenzo-p-dioxins, *PCDFs* Polychlorinated dibenzofurans

DEHP is the most ubiquitous phthalate in the environment. There are already many studies about is disrupting activity in the reproductive female system [56] evaluated complex factors, after administration during 14 days of 40 µg/kg body weight. This exposure resulted in expression problems, represented in Fig. 3, in actin and Juno protein, the morphology of the spindle meiotic altered, also resulted in oxidative stress and apoptosis, the ATP content and mitochondria were also affected, DNA destruction, and epigenetic modifications in DEHP-exposed mouse oocytes.

The results obtained confirm the disruptive activity that DEHP can cause on these structures, leading to the conclusion that this compound can diminish maturation and fertilization on oocytes. Disturbances in numerous processes as steroidogenesis that affect the hypothalamic-pituitary-ovarian axis formation were also found after the exposure in the mouse ovary [69].

**Fig. 4:**
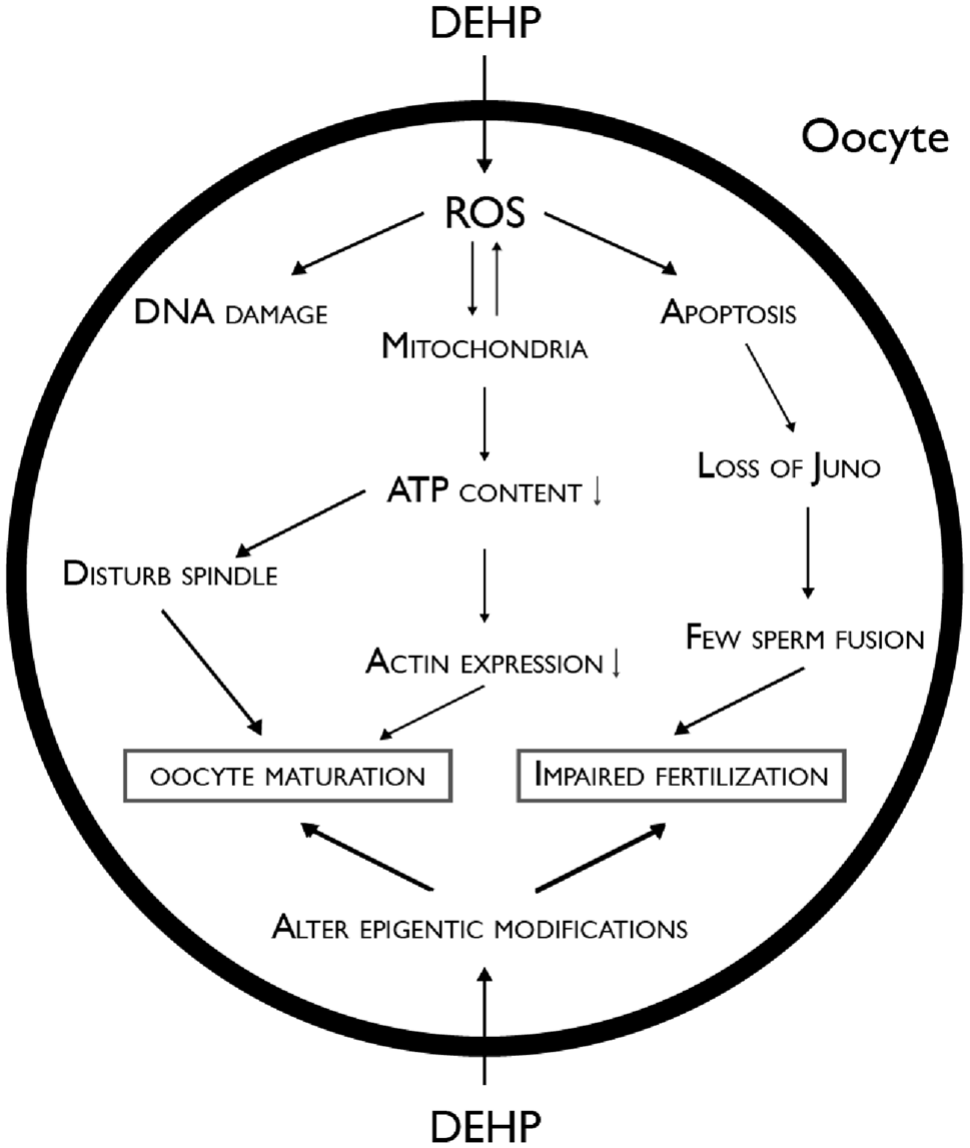
Comprehensive explanation about the possible mechanisms of action of DEHP; *DEHP* Bis(2-ethylhexyl) Phthalate. Adapted from [50]

A prospective cohort study carried out between 2004 and 2012 analyzed two urine samples per cycle before oocyte retrieval from 256 women. Eleven phthalate metabolites (MEHP, MEHHP, MEOHP, MECPP, MiBP, MBP, MBzP, MEP, MCOP, MCNP, and MCPP) were measured. These urinary samples were evaluated in different models and women with the higher DEHP metabolites were directly associated with diminished oocyte production, clinical pregnancy, and live birth [70]. The Fig. 4 shows a comprehensive explanation about the possible mechanisms of action of DEHP; DEHP: Bis(2-ethylhexyl) Phthalate.

**Fig. 5:**
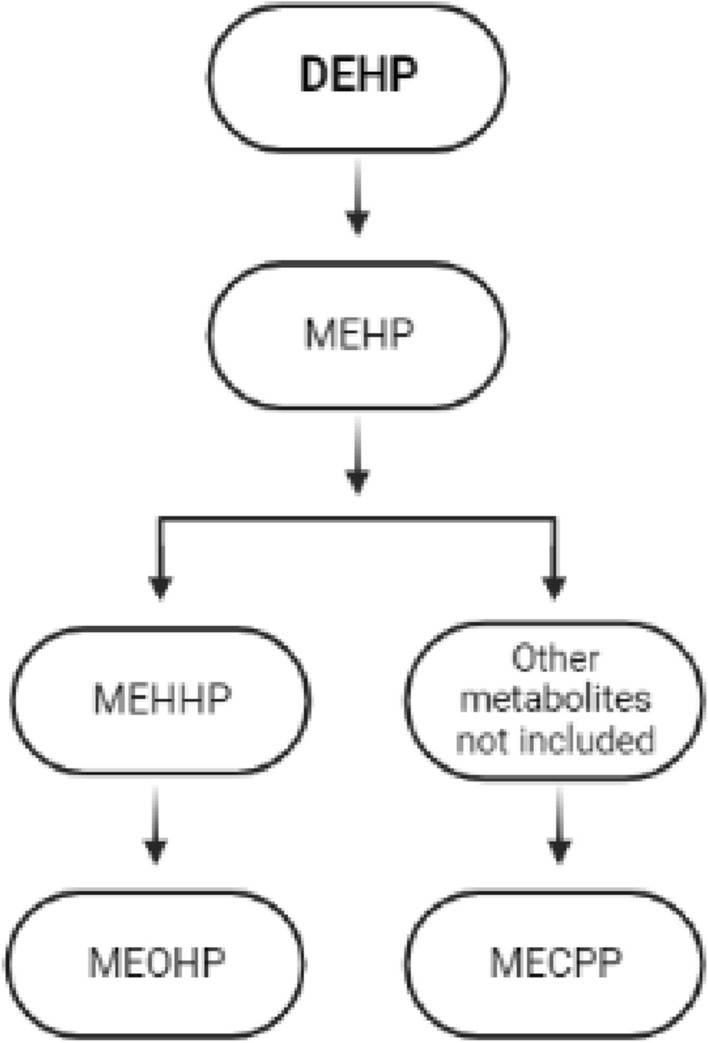
Metabolites of DHEP. Adapted from [62]. *DHEP* di(2-ethylhexyl)phatalate, *MEHP* mono(2-ethylhexyl)phthalate, *MEHHP* 2-ethyl-5-hydroxy-hexylphthalate, *MEOHP* 2-ethyl-5-oxy-hexylphathalate, *MECPP* 2-ethyl-5-carboxy-pentylphthalate

The objective of the cross-sectional study was to use a nationally representative sample from the 2013–2016 National Health and Nutrition Examination Survey (NHANES) data to assess the potential association between DEHP exposure and infertility in women, therefore determinate urinary phthalate metabolite levels as biomarkers for DHEP exposure were associated with infertility. Data was only available in women ages 20–44. The study starts with 1199 participants, but final study population was 975 and the measurement of infertility was assessed with response of two questions: “Have you ever attempted to become pregnant over a period of at least a year without becoming pregnant?” and “Have you ever been to a doctor or other medical provider because you have been unable to become pregnant?”. Any women who responded “Yes” to either of these questions was considered to have a history of infertility. They examinated four metabolites of DHEP (Fig. 5): mono (2- ethylhexyl)phthalate (MEHP), mono(2-ethyl-5-hydroxy-hexyl) phthalate (MEHHP), mono(2-ethyl-5-oxy-hexyl) phthalate (MEOHP), and mono(2-ethyl-5-carboxy-pentyl)phthalate (MECPP). Phthalates, including DEHP, have short half-lives in humans and are excreted as monoester metabolites in urine, making these appropriate biomarkers of DEHP exposure. In this study, they don´t find any association between DHEP metabolites and reduced fecundity. And metabolites of DHEP were associated with reduced probability of clinical pregnancy and live birth. They reinforced that are several limitations in their study, like the measurement of exposure to DHEP is limited due to the fact that it does not bioaccumulate and has a short half-life in the body and they cannot rule out potential confounding variables thar were either unmeasured or not included in the analysis [71].

Together, these data raise concerns about the impacts of chemicals on female reproductive health. There is still a data gap that needs to be filled with more studies on this field, epidemiological studies, in vivo and in vitro studies, and environmental studies to clarify all the exposure routes to human health. Nonetheless is evident the association between this class of chemicals environmental disruptors and female infertility, and the need for public awareness on this subject is urgent as well as the right protection against it.

### Pesticides: organochlorine and organophosphate compounds

The use of pesticides has been growing as mentioned above. The purpose was to improve agriculture, but not only affected the crop but also altered the food chain and the ecosystem. OCPs are a group of chlorinated compounds, widely used as pesticides, and known for their high persistence in the environment. Dietary exposure to OCPs and their damages to reproductive health is now the highlights of the research, they bioaccumulate and are toxic to humans and wildlife. They can be found among food items, fatty food and dairy products [72, 73].

**Fig. 6:**
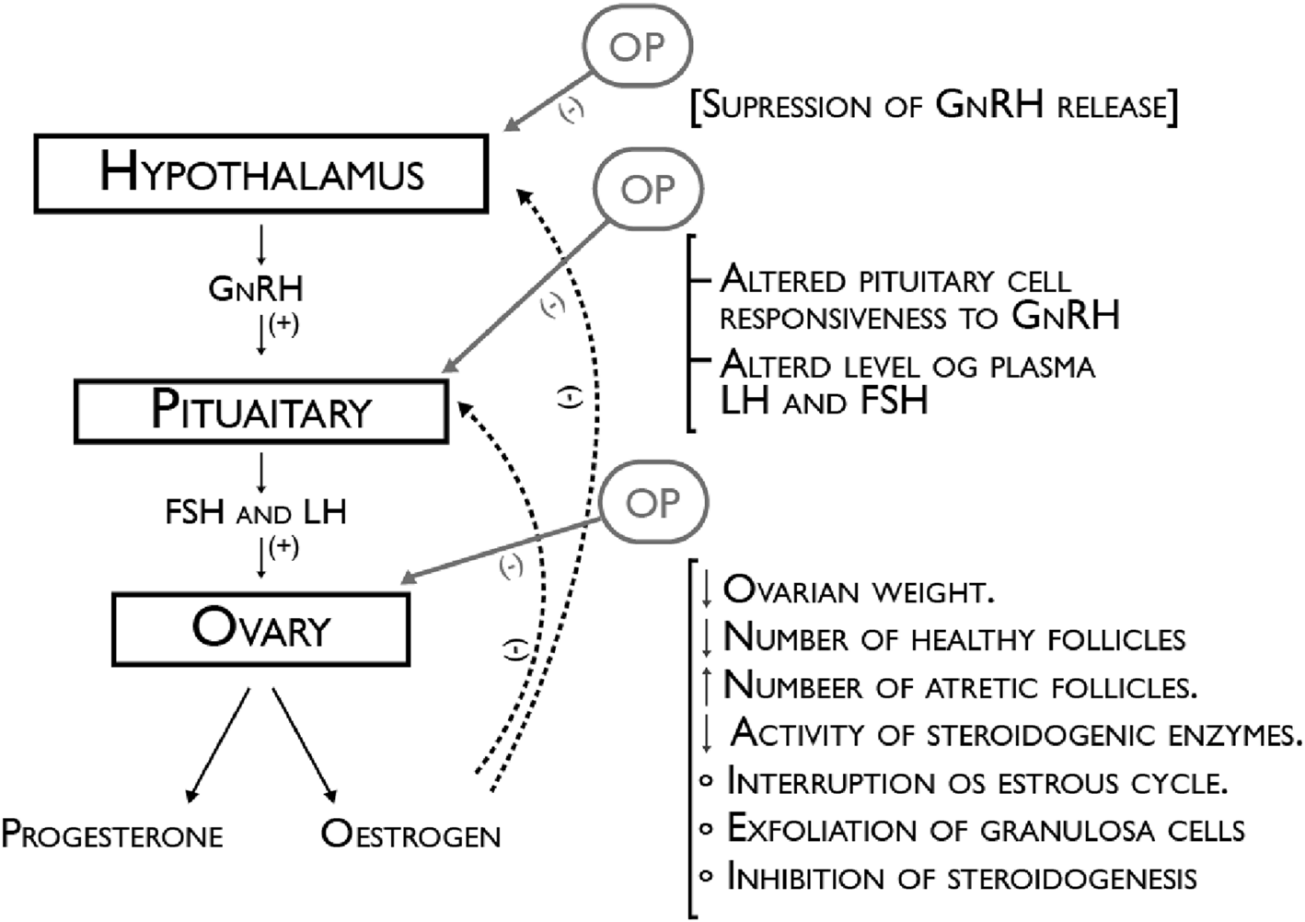
Schematic explanation of the OP potential target sites of action in the regulation of reproductive female functions through the HPG axis; *OP* Organophosphate pesticides, *GnRH* Gonadotropin-releasing hormone, *FSH* Follicle-stimulating hormone, *LH* Luteinizing hormone. Adapted from [75]

Another group of pesticides called organophosphate pesticides (OPs) has replaced some groups of organochlorines due to its fast degradation, a lower accumulative potential in the environment and animal tissues, a diminished possibility to enter to the ecosystem through food, and a greater selective process on causing toxicity only among insects and not on other vertebrates. In Fig. 6 are a schematic explanation of the OP potential target sites of action in the regulation of reproductive female functions through the HPG axis [74].

However, studies present in Table 6 [76-78] and 7 [79] reported their severe toxicity and pointed out some OPS as probable disruptors on the reproduction physiology, even at very low doses. Impaired functions as processes of HPG axis are the result suspected by the release of neurotransmitters by this class of pesticides, being this the mechanisms by which OPs interfere with the reproductive health system [74].

**Table 6:** Observational studies of pesticides found in food

Participants/subjects and research design	Patients	Patient parameters	Outcomes/main conclusion of the study	References
Examine breast milk OCPs and their associations with female reproductive function	68 women	Breast milk	Dietary habit is an important factor influencing the levels of OCPs in breast milk and the associated risks for women	[70]
Examine the association of preconception intake of pesticide residues in FVs with outcomes of infertility treatment with ART	325 women	FVs items in the FFQ and PDP, and Corresponding Scores for First, Second, and Third Measure, and PRBS	Higher consumption of high–pesticide residue FVs was associated with lower probabilities of pregnancy and live birth following infertility treatment with ART	[71]
To see if there is an association of serum levels of typical organic pollutants with PCOS	50 women with PCOS and 30 normal controls	Serum levels	The PCOS group showed higher serum levels of PCBs, PAHs, and pesticides than the control group.	[72]

*OCPs* Organochlorine pesticides, *FVs* Fruits and vegetables, *ART* Assisted reproductivetechnologies, *FFQ* Food Frequency Questionnaire, *PDP* Pesticide Data Program, *PRBS* Pesticide Residue Burden Score, *PCOS* Polycystic ovary syndrome, *PCBs* Polychlorinated biphenyls, *PAHS* Polycyclic aromatic hydrocarbons

**Table 7:** *In vivo* study with diazinon

Research design	Dosage regimen	Parameters monitored	Outcomes/main conclusion of the study	References
To evaluate DZN effects on apoptosis of ovarian follicles in adult rats and also to assess the protective role of vit. E	Experimental group 1 (DZN+olive oil, 60 mg/kg), experimental group 2 (vit E, 200 mg/kg), and experimental group 3 (DZN+vit E, the same dosage)	Measure apoptosis of ovarian follicles	The number of apoptotic cells in experimental group 1 increased significantly in the contrast control group in secondary and graafian follicles. Administration vit E plus DZN, significantly reduced apoptotic cells compared to DZN group	[73]

*DZN* Diazinon

In Taiwan, a study was conducted to examine OCPS in breast milk, and their association with female reproductive function, making connections with other parameters such as dietary and sociodemographic factors. 68 samples of breast milk were examined, and DDT and Hexachlorocyclohexane were the most abundant OCPs. Also, the relation between the presence of OCP residues in the breast milk and factor such as family income, cow milk, and beef intakes were positive. This study allows concluding about the tight connection between dietary habits and the exposure levels of OCPs [76].

With the aim of investigating the possible outcomes of infertility treatment, when associated with the intake of pesticides present in fruits and vegetables, a study was conducted in a group of 325 women, under 541 assisted reproductive technologies at a fertility center. The exposure regime was divided into two groups of high and low exposure to pesticide residues present in fruits and vegetables, based on questionnaires performed on the woman involved. With this, it was possible to associate the consumption of higher levels of pesticides with lower probabilities of achieving clinical pregnancy and live birth. On the other hand, no significant relations between low pesticide residues were made. This data suggests that dietary pesticide exposure, in a range of typical daily consumption, may be associated with adverse reproductive consequences [77].

A preliminary case-control study undertaken at a reproductive Center with 50 women affected by PCOS, was taken upon the question if there is an association between this clinical condition and serum levels of organic pollutants. The results of this study take us to the positive relation between PCOS and organic pollutants, since the serum levels were substantially higher in PCOS group when compared to the control one, especially DDE and polychlorinated biphenyls [78].

In another study, they wanted to focus specifically on fertility in women and investigated associations between chemical exposures and ovarian reserve. Thereunto they used a cohort pregnant woman undergoing caesarean section (n = 145) was recruited at Karolinska University Hospital Huddinge in Stockholm, Sweden, during years 2015–2018. To analyse the effects of environmental chemicals on ovaries. They measured nine OCPs, between others persistent organic pollutants. The result of the study shows that exposure to chemicals may reduce the size of ovarian reserve in humans, and they strongly encourage new studies for infertility in women in more detail [73].

Epidemiological studies connected in China have demonstrated relatively high levels of exposure to OCPs in women, raising concerns about chronic exposures and potential effects on reproductive health. In this study they aimed to prospectively evaluate the associations between preconception pesticide exposure and couple fertility in Shangai, China, where OCPs are the most widely used pesticides, and confirmed that affect time to pregnancy (TTP) and couple fertility [29].

DZN is another concerning organophosphate insecticide extensively used in agriculture that is responsible for many negative effects on humans, including reproductive ones. This study evaluated the effects of DZN on apoptosis of ovarian follicles in adult rats and also assessed the possible protective role of vitamin E. Thirty adult female rats were divided into groups, control, sham, and two experimental (one with DZN + olive oil, another with only vit.E and DZN + vit. E). As expected, DZN demonstrated apoptotic activity in secondary follicles (group with DZN + olive oil), and another interesting data was that the group DZN + vit. E exhibited a protective role on DZN toxicity [79]. This compound is also pointed as an inducer of oxidative stress, in tissues such as the reproductive system. Other studies had already approached the protective role that this vitamin can display, showing some interesting data on this concern [80].

The interest within the general population on this matter has been rising in the last decades. A growing concern in the search for organic products, free from pesticides, antibiotics, and genetically modified organisms has been observed. In general terms, the awareness on this topic has been growing, and in this line a more conscious attitude about environmental contamination which leads to a higher demand for organic products [81, 82].

There are already many different studies on the benefits of the consumption and preference for organic food, as well studies that compare the exposure of pesticides, through the analyses of the urine, that confirms the positive relationship between consumption of organic food and lower pesticides metabolites in the urine [83, 84].

### Dioxins and dioxin-like compounds

Major concern over the chemical group of dioxins has arisen since the discovery of the highly toxic and teratogenic TCDD. They belong to the persistent organic pollutants and act via a common mechanism that consists of stimulating the receptor of aryl hydrocarbon. The main source of dioxins in animals and humans is through food, and due to their highly lipophilic profile, they tend to accumulate in tissues with a high-fat percentage. The metabolism and excretion of these chemicals are generally extremely slow [85, 86].

Some studies (Table 8, [16, 87]) have investigated the correlation between the serum levels of dioxin-like substances in patients with endometriosis. Endometriosis is an estrogen-dependent disease of the reproductive feminine health tract [42, 43]. Adipose tissue from patients diagnosed with endometriosis was analyzed and compared to a control group, to see if there was a positive correlation with the presence of dioxin-like substances. Their results suggest a relation between this exposure and this female disorder [87, 88].

**Table 8:** Observational studies with dioxins found in food

Participants/subjects and research design	Patients	Patient parameters	Outcomes/main conclusion of the study	References
Examined relationships of TCDD exposure with TTP (TTP, the monthly probability of conception within the first 12 months of trying) and infertility (12 months of trying to conceive)	981 women exposed to TCDD in a 1976 accident	Serum TCDD concentration and estimated TCDD concentration at pregnancy	TCDD exposure may be associated with decreased fertility in Seveso mothers and potentially in their daughters exposed in utero	[18]
To study the levels of biologically active dioxin-like substances in adipose tissue of patients with DIE	30 patients	Dioxin-like substances were analyzed in adipose tissue	The total toxic equivalence and concentrations of both dioxins and PCBs were significantly higher in patients with DIE	[83]

*TCDD* 2,3,7,8-tetrachlorodibenzo-p-dioxin, *TTP* Time to pregnancy, *DIE* deep infiltrating endometriosis, *PCBs* Polychlorinated biphenyls

Since Seveso’s incident in 1976, a huge study called Seveso Women’s Health Study (SWHS) was created to follow 981 women that were exposed to TCDD. In the last report of this study population, the median factor time to pregnancy (TTP) on SWHS women was 3 months, but 18% reported TTP ≥ 12 months. On other hand, SWHS daughters had different results, their median TTP was 2 months, and 11% reported taking more than 12 months to conceive. The results expressed an association between TCDD exposure and decreased fertility and probably exposure *in utero* [16].

To investigate the effect of TCDD exposure on maternal ovaries, a study summarized in Table 9 [88, 89] was performed on pregnant rats treated with TCDD with different doses (100 and 500 ng/kg), contrasting with a control group (corn oil). Ovary weight, E2 and FSH concentrations, estrous cycles, and the number of follicles were studied factors that expressed changes. The results obtained in this study demonstrated that the exposure in utero of TCDD, especially the highest exposure level, may display a significant change in the parameters evaluated, leading us to its possible negative role on the ovary development and its functions. It was also made a positive relation between the down-regulation of mRNA and protein expression with these outcomes [89].

On the same matter, but studying the transgenerational impact on F3, another study assessed the TCDD exposure and ancestral effects on ovarian toxicity, directing their attention to the Igf2/H19 pathway, an important route for follicular development. F0 pregnant rats were divided, and some received the TCDD in 100 or 500 ng/kg BW/day and others vehicle as part of the control, through 8–14 days of gestation. The results indicated a decrease in the ovarian coefficient, LH concentration, number of primary follicles, and a rise in the apoptosis of granular cells was also registered. Through an RT-PCR analysis, it was possible to record an increased level of expression on H19 mRNA in ovaries treated with F3. All this data led us to the transgenerational impairment on the adult ovary enhancement and functions when exposed to TCDD and possibly bound to an Igf2/H19 inhibition [91].

**Table 9:** *In vivo *studies with dioxins found in food

Research design	Dosage regimen	Parameters monitored	Outcomes/main conclusion of the study	References
To investigate the effect of maternal exposure to TCDD on ovaries	TCDD (100 ng/kg or 500 ng/kg) or only vehicle and corn oil	The vaginal opening and estrous cycle of female offspring rats were monitored twice a day. The ovarian histology, follicle counts, real-time PCR, western blotting, and DNA methylation analysis about Gdf9 and Bmp15 were carried out in F1 rats	Maternal exposure to TCDD could affect the ovary development and functions which were possibly associated with down-regulation of mRNA and protein expression of GDF9 and BMP15	[87]
The aim of our study was to evaluate the effect of ancestral TCDD exposure on ovarian toxicity in offspring rats (F3), focusing on the Igf2/H19 pathway	100or 500 ng/kg BW/day	Ovary coefficient; Vaginal opening time; Regularity of estrous cycle; Ovarian pathology; Follicles counts; Apoptosis of granular cells; Levels of E2, FSH, and LH	Our data showed that ancestral TCDD exposure may impair transgenerational adult ovary development and functions	[89]

*TCDD* 2,3,7,8-tetrachlorodibenzo-p-dioxin, *PCR* Polymerase chain reaction, *BW* Bodyweight, *E2* 17β-Estradiol,* FSH* Follicle-stimulating hormone, *LH* Luteinizing hormone

Equine gametes were also investigated to study the effect of TCDD exposure (0.32 ng, 3.2 ng, and 32 ng/mL). 28.38% of the oocytes under the lowest concentration reached the oocyte maturation and on the 3.2 ng/mL the percentage was reduced to 5.14%. The highest concentration in the study was 32 ng and non-matured oocytes were observed. This came to add evidence of the negative impact on the female reproductive system [92].

The awareness on this matter is urgent. There is still a lot to study and ascertain about the effects on reproductive health coming from dioxin exposure. It is clear that there are intrinsic harmful effects, but the mechanism behind it and all the physiological processes within are still very unclear. There are a lot more studies connecting this chemical family with male reproductive health when compared to females, and even with a lot more biography uncertainties still exist.

## Conclusions

In the last two decades, there has been a growing awareness of endocrine-disrupting chemicals and their possible adverse effects on human health. This subject is considered a severe health problem worldwide, as well as infertility. Data from WHO revealed that nowadays about 48 million couples and 186 million individuals suffer from infertility around the globe. Where these two subjects meet is determined by the intimate role that these endocrine disruptive chemicals play in female and male infertility, being the female infertility the focus of this study [93].

During the past 6 years, several studies examined the effects of endocrine disrupting chemicals on female fertility. Collectively, these studies showed that the compounds that are most frequently related and responsible for regarding female fertility are plasticizers, particularly BPA and phthalates, such as DEHP and its metabolites, organochlorine, and organophosphate compounds, namely diazinon, and dioxins and dioxin-like compounds. The first two groups interfere with different processes within ovarian development, such as folliculogenesis, steroidogenesis, development of the female germline stem cells, follicle formation, and also present strong associations with diseases such as PCOS. The imbalance of the HPO axis is another issue pointed out for the failure of the reproductive tract, generating disturbances in hormone homeostasis.

Organochlorine and organophosphate compounds have also been shown to affect ovaries parameters as well as the mentioned above. They have been proven to induce follicles apoptosis and display impaired processes on the HPO axis. Associations between this family of chemicals and ovaries diseases, such as PCOS, were also established.

At last, dioxins and dioxin-like compounds are also related to female infertility, however, there is a lot to explore in this group, especially related to female health issues. The most presented compound throughout the research was TCDD, which was shown to have a clear negative impact on the female reproductive tract, displaying threats to ovarian function and other related diseases for example endometrioses. The TTP was also reduced when exposed to this chemical, which is another indicator of the negative role of the biological processes regarding reproduction.

The endocrine system is complex, which consists of one difficult barrier on the discovery of the different mechanisms around these compounds. Whereas this exposure can trigger failures on the reproductive system or not, has created a credible and alarming question. The biological complexity behind it is still to discover and clarify. The present literature review revealed that despite all the quality work involving all the scientific community, a lot more is needed to solve this complex and urgent subject that created a major threat to Human Health. In this line, large, double-blind, placebo-controlled randomized clinical trials are needed to better understand the mechanisms of action of these compounds in female infertility, as well as the doses and frequency of exposure responsible for it. It would also be of great value to know which compounds (e.g. vitamins) can play a protective effect regarding the Human Health damages caused by endocrine disruptors.

## Supplementary information

Below is the link to the electronic supplementary material.

Supplementary material 1 (DOCX 120.8 kb)

## Data Availability

Not applicable.

## Abbreviations

AhR: Aryl-hydrocarbon receptor
AR: Androgen receptor
BPA: Bisphenol A
BPE: Bisphenol E
BPF: Bisphenol F
BPS: Bisphenol S
DBP: Di-n-butyl phthalate
DDT: Dichlorodiphenyltrichloroethane
*DEHP*: Di(2-ethylhexyl) phthalate
DHA: Docosahexaenoic acid
DiBP: Di-iso-butyl phthalate
DZN: Diazinon EDs
EDCs: Endocrine-disrupting chemicals
EFSA: European Food Safety Authority
EPA: Eicosapentaenoic acid
ER: Estrogen receptor
FGSCs: Female germline stem cells
FSH: Follicle-stimulating hormone
GnRH: Gonadotropin-releasing hormone
HPG: Hypothalamic–pituitary–gonadal
HPO: Hypothalamic-pituitary-ovarian
IARC: International Agency for Research on Cancer
LH: Luteinizing hormone
MBP: Mono-n-butyl phthalate
MBzP: Monobenzyl phthalate
MCNP: Monocarboxyisononyl phthalate
MCOP: Monocarboxyisooctyl phthalate
MCPP: Mono(3-carboxypropyl) phthalate
MECPP: Mono(2-ethyl-5-carboxypentyl) phthalate
MEHP: Mono-2-ethylhexyl phthalate
MEOHP: Mono(2-ethyl-5-oxohexyl) phthalate
MEP: Monoethyl phthalate
MiBP: Mono-isobutyl phthalate
NIH: National Institutes of Health
OCPs: Organochlorine pesticides
OPs: Organophosphate pesticides
PCBs: Polychlorinated biphenyls
PCDDs: Polychlorinated dibenzo-p-dioxins
PCDFs: Polychlorinated dibenzofurans
PCOs: Polycystic ovary syndrome
POPs: Persistent organic pollutants
PR: Progesterone receptor
PVC: Polyvinyl chloride
SML: Specific migration limit
SWHS: Seveso Women’s Health Study
TCDD: 2,3,7,8-Tetrachlorodibenzo-p-dioxin
TTP: Time to pregnancy
WHO: World Health Organization
