# Supplementary material for: The role of endocrine disruptors in female infertility
Source: Mol Biol Rep. 2023 Jul 4;50(8):7069–88. doi: 10.1007/s11033-023-08583-2 (PMC10374778; doi:10.1007/s11033-023-08583-2)
Supplement: Supplementary file 1 — Supplementary material 1 (DOCX 120.8 kb) [file 11033_2023_8583_MOESM1_ESM.docx]

Supplementary Material

**Table 1A.** Classifications, chemical properties, and sources of the most common EDs.

| **EDC** | **General Chemical Structure** | **Group** | **Route of exposure** | **Sources** | **Half-life** |
| --- | --- | --- | --- | --- | --- |
| BPA | 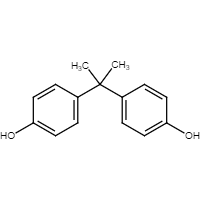 | Bisphenols/ Plasticizers | Ingestion, inhalation, dermal absorption | Polycarbonate plastics, thermal paper, epoxy resins, plastic toys, and bottles, lining of food cans. | 4-5 hours |
| DEHP | 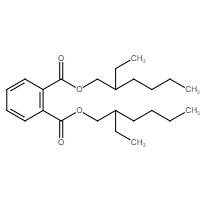 | Phthalates/ Plasticizers | Ingestion, inhalation, dermal absorption | Medical devices, artciles made of PVC. | 4-8 hours |
| DDT | 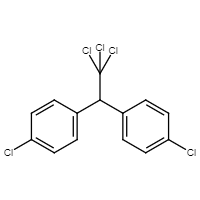 | Organochloride/ Pesticides | Ingestion, inhalation, dermal absorption | Contaminated water, soil, fish. | 6-10 years |
| TCDD | 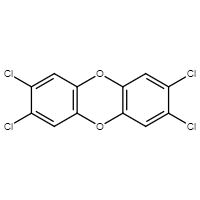 | Dioxin | Ingestion, inhalation | Combustion of fossil fuels, incineration processes. | 1.6-3.2 years |

**Note:** BPA: Bisphenol A; DEHP: Di(2-ethylhexyl) phthalate; DDT: Dichlorodiphenyltrichloroethane; TCDD: 2,3,7,8-tetrachlorodibenzo-p-dioxin; PVC: Polyvinyl chloride.

**Table 2A.** Principal phthalates that had been demonstrated to interact with estrogen receptor and progesterone receptor (PR) in humans. Data extracted from [14].

| **Parent Phthalates**  **(Phthalate-diester)** | **Primary Metabolites**  **(Phthalate-monoester)** |
| --- | --- |
| DEHP  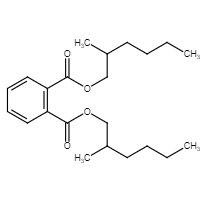 | MEHP  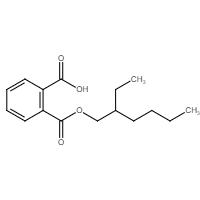 |
| DiBP  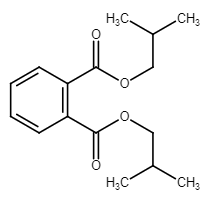 | MiBP  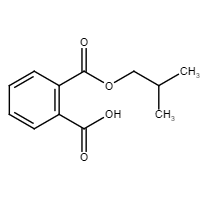 |

**Note:** DEHP: Di(2-ethylhexyl) phthalate; MEHP: Mono-2-ethylhexyl phthalate; DiBP: Di-iso-butyl phthalate; MiBP: Mono-iso-butyl phthalate.

**Table 3A.** The most common organochlorine and organophosphate compounds, their chemical structure, use, and persistence. Adapted from [28].

| **Chemical name** | **IARC group** | **Use** | **Persistence in environment** | **WHO classification based on rat oral LD50** |
| --- | --- | --- | --- | --- |
| DDT | Group 2A | Acaricide insecticide | High persistence  Half-life: 2-15 years | Moderately hazardous |
| DDD | - | Insecticide | High persistence  Half-life: 5-10 years | Acute hazard in unlikely |
| DDE | - | Insecticide | High persistence  Half-life: 10 years | Slightly hazardous |
| Diazinon | Group 2A | Insecticide | Moderately persistence  Half-life: 37-38 days | Moderately hazardous |

**Note:** DDT: Dichlorodiphenyltrichloroethane; DDD: Dichlorodiphenyldichloroethane;

DDE: Dichlorodiphenyldichloroethylene.

**Table 4A.** Observational studies of plasticizers and fertility-related problems.

| **Participants/subjects and research design** | **Patients** | **Patient parameters** | **Outcomes/Main conclusion of the study** | **References** |
| --- | --- | --- | --- | --- |
| Investigate the role of BPA in the pathogenesis of PCOS and other metabolic parameters | 112 girls with PCOS and 61 controls | Serum BPA and oral glucose tolerance test | Adolescents with PCOS presented higher BPA concentrations than controls and there was a significant relation with androgen levels | [53] |
| To investigate possible associations between reproductive hormone levels among woman exposed to BPA | 106 women exposed and 250 unexposed | Blood samples to analyze: FSH, LH, E2, PRL and PROG; Urine samples for BPA measurement | Evidence of disruptive activity of BPA on women's hormone homeostasis were found | [62] |
| Evaluate serum levels of BPA in exposed women with PCOS and hormonal and metabolic effects | 62 women with PCOS and 62 healthy women | Serum samples to analyze BPA; Fasting blood; Triglyceride; Cholesterol HDL and LDL; TSH concentration and LH:FSH ratio | BPA levels were higher in BPA exposed PCOS women than the group of healthy women. Major differences in the other metabolic parameters | [52] |
| Search the presence of eight phthalate metabolites on women attending an infertility clinic and its possible correlations | 112 women | Urine samples per cycle to measure 11 urinary phthalate metabolites | DEHP and DiDP concentrations were inversely associated with oocyte yield and number of matured oocytes at retrieval; DiNP and DiDP were associated with reduced fertilization; DEHP metabolites were negatively associated with probable clinical pregnancy and live birth following IVF | [63] |
| Study the concentrations of 8 phthalate metabolites | 112 women attending an infertility clinic | Follicular fluid and urine samples | Most of the studied phthalates were highly detected in the ovarian follicular fluid of women undergoing IVF despite in lower doses than those shown to induce ovarian toxicity in animal studies | [64] |

**Note:** BPA: Bisfenol A; PCOS: Polycystic ovary syndrome; FSH: Follicle-stimulating hormone; LH: Luteinizing hormone; E2: 17β-Estradiol; PRL: Prolactin; PROG: Progesterone; TSH: Thyroid stimulating hormone; DEHP: Di(2-ethylhexyl) phthalate; DiDP: Di-isodecyl phthalate; DiNP: Di-isononyl phthalate; IVF: *in vitro* fertilization.

**Table 5A.** Observational studies of pesticides found in food.

| **Participants/subjects and research design** | **Patients** | **Patient parameters** | **Outcomes/Main conclusion of the study** | **References** |
| --- | --- | --- | --- | --- |
| Examine breast milk OCPs and their associations with female reproductive function | 68 women | Breast milk | Dietary habit is an important factor influencing the levels of OCPs in breast milk and the associated risks for women | [73] |
| Examine the association of preconception intake of pesticide residues in FVs with outcomes of infertility treatment with ART | 325 women | FVs items in the FFQ and PDP, and Corresponding Scores for First, Second, and Third Measure, and PRBS | Higher consumption of high–pesticide residue FVs was associated with lower probabilities of pregnancy and live birth following infertility treatment with ART | [74] |
| To see if there is an association of serum levels of typical organic pollutants with PCOS | 50 women with PCOS and 30 normal controls | Serum levels | The PCOS group showed higher serum levels of PCBs, PAHs, and pesticides than the control group. | [75] |

**Note:** OCPs: Organochlorine pesticides; FVs: Fruits and vegetables; ART: Assisted reproductive
technologies; FFQ: Food Frequency Questionnaire; PDP: Pesticide Data Program; PRBS: Pesticide Residue Burden Score; PCOS: Polycystic ovary syndrome; PCBs: Polychlorinated biphenyls; PAHS: Polycyclic aromatic hydrocarbons.

**Table 6A:** *In vivo* study with diazinon

| **Research design** | **Dosage regimen** | **Parameters monitored** | **Outcomes/Main conclusion of the study** | **References** |
| --- | --- | --- | --- | --- |
| To evaluate DZN effects on apoptosis of ovarian follicles in adult rats and also to assess the protective role of vit. E | Experimental group 1 (DZN+olive oil, 60 mg/kg), experimental group 2 (vit E, 200 mg/kg), and experimental group 3 (DZN+vit E, the same dosage) | Measure apoptosis of ovarian follicles | The number of apoptotic cells in experimental group 1 increased significantly in the contrast control group in secondary and graafian follicles. Administration vit E plus DZN, significantly reduced apoptotic cells compared to DZN group | [76] |

**Note:** DZN: Diazinon.

**Figure 1A.** Example of an analogy of the chemical structures of the natural estrogen hormone estradiol and bisphenol A, a xenoestrogen endocrine disruptor.

| 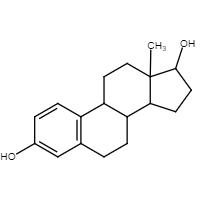 | 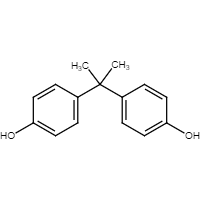 |
| --- | --- |
| Estradiol | Bisphenol A |

| 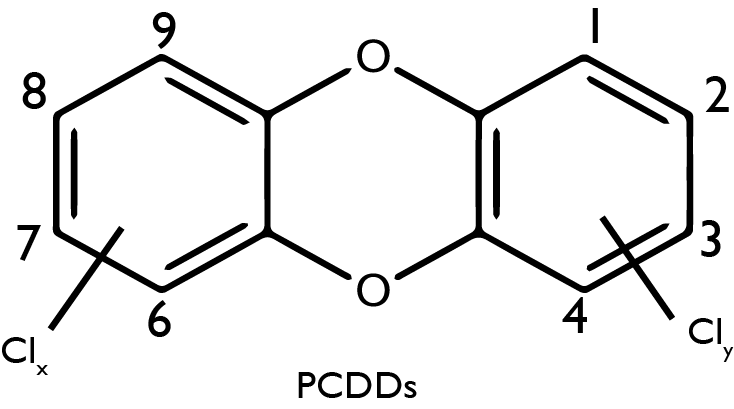 | 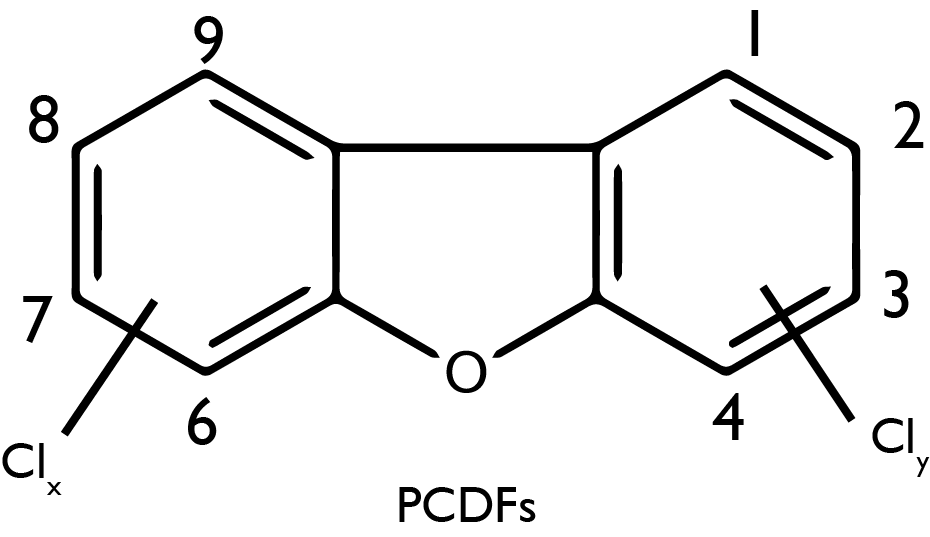 |
| --- | --- |

**Figure 2A.** Structure of dioxins (PCDDs and PCDFs). Relevant PCDD/Fs are substituted with additional chlorines at positions 2,3,7 and 8; PCDDs: Polychlorinated dibenzo-p-dioxins; PCDFs: Polychlorinated dibenzofurans.


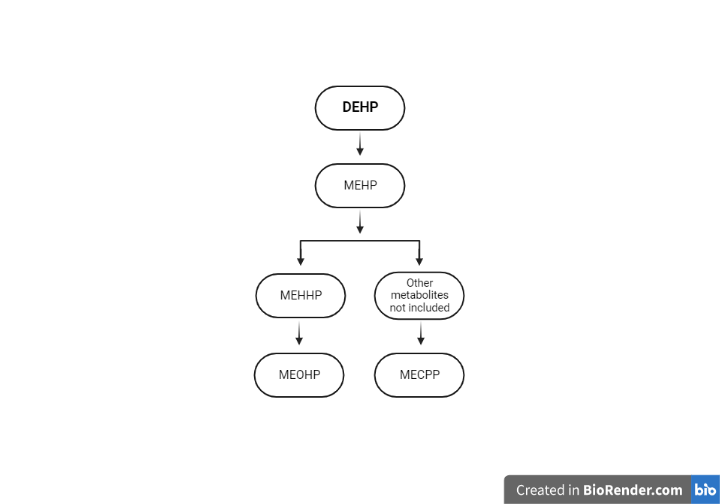
**Figure 3A.** Metabolites of DHEP. Adapted from [68]

**Note:** DHEP: di(2-ethylhexyl)phatalate; MEHP: mono(2-ethylhexyl)phthalate; MEHHP: 2-ethyl-5-hydroxy-hexylphthalate; MEOHP: 2-ethyl-5-oxy-hexylphathalate; MECPP: 2-ethyl-5-carboxy-pentylphthalate.
